# The Growth Pattern of Tibetan Infants at High Altitudes: a Cohort Study in Rural Tibet region

**DOI:** 10.1038/srep34506

**Published:** 2016-10-03

**Authors:** Weihua Wang, Feng Liu, Zhicheng Zhang, Yi Zhang, Xiaojing Fan, Ruru Liu, Shaonong Dang

**Affiliations:** 1 Shaanxi Center for Disease Control and Prevention, No. 3 Jian Dong Street, Xi’an, Shaanxi, People’s Republic of China; 2 The Department of Epidemiology and Biostatistics, School of Public Health, Xi’an Jiaotong University Health Science Center, Xi’an, No. 76, Yanta West Road, Xi’an, Shaanxi, People’s Republic of China; 3 Xi’an Center for Disease Control and Prevention, Shaanxi, No. 599, Xiying Road, Xi’an, Shaanxi, People’s Republic of China

## Abstract

Studies on growth pattern of Tibetan infants and the difference from other child groups were limited due to its special living environment and unique customs. In this study, 253 Tibetan infants were followed-up from their birth to 12^th^ month in rural Tibet. Five visits were conducted and weight and length were measured at each visit. Mixed model was employed to analyze the growth pattern of Tibetan infants and its comparison to the Han infants. Propensity Scores (PS) technique was adopted to control for the potential confounding factors. The mixed model found that the birth weight/length had a negative impact on the increment of Tibetan infants after birth (weight: *β* = −0.6819, *P* < 0.0001, length: *β* = −0.9571, *P* < 0.0001). The weight increment of Tibetan infants was greater than Han infant with age (*β*_age*ethnic_ = 0.0345, P < 0.001), after using PS as a covariant. And another mixed model in which PS was used as a matching factor found similar trend. Compared with Chinese Han infants, Tibetan infants were lower weight and shorter length within one year after birth but they had greater increment of weight, suggesting that Tibetan infants might have a significant catch-up growth within the first year of life.

Size at birth and early infancy growth had long been recognized to be important indicators of maternal and offspring health, and of early childhood survival^1^,^2^. A lot of surveys showed that the weight and length of Chinese infants had increased with its economic growth, especially the last 30 years, in which the urban infants had got to the standard of World Health Organization (WHO) made in 2006^3^,^4^,^5^. Tibet is located at Qing-Tibetan highland with high altitudes and Tibetan people usually live in high-altitude hypoxia environments with average altitude of 4000 m^6^. There had already been some studies on Tibetan children, due to its special living environment and unique customs, while most of them focused on evaluation of nutritional status by height and weight^7^,^8^,^9^, feeding practice^10^, or their adaption to high altitude^11^. However, the studies on their own growth pattern and the difference from other child groups were limited, especially based on longitudinal study data. In this study, we tried to describe the basic growth pattern of Tibetan infants living at high altitudes by following up them from birth to 12^th^ month and compared their growth pattern with that of Chinese Han children living in the plain.

## Subjects and Methods

### Study setting

Tibet is located on the highest plateau in the world, where 86% of the area is at an altitude of >4000 m and the typical environmental features was hypoxic and cold climate. The special geographical environment has constructed the unique Tibetan cultural and social environment. Farming and animal husbandry was the main economic activities. The rural population account for 87% of the total population and >95% of people belong to the Tibetan ethnic group^6^,^12^. The economic status of Tibet has been improved much. The per capital income of rural residents increased from 3176.0 RenMinBi Yuan (RMB) in 2008 to 4904.4 RMB in 2011^13^. The study was conducted in Lhasa area with average altitude of 3600, which is the capital of Tibet with typical Tibetan culture and also one of the main populated area in Tibet. Two counties of Lhasa were selected as study area. Six townships from Dazi county had a mean altitude of 3,685 m and five townships from Qushui county with a mean altitude of 3,500 m.

### Study design and subjects

A cohort study design was adopted in this study. The Tibetan infants born in the project of the multi-micronutrients supplementation during pregnancy in Tibet were included as the subjects. The project’s objective was to improve nutritional status of Tibetan pregnant women and birth outcome and it was conducted in Dazi and Qushui county of Lhasa area. The two counties were selected on the basis of available information on socio demographic factors, population size, and prevalence of childhood malnutrition. Both counties shared many similar characteristics with other rural areas of Tibet and did not present any exceptional features. Considering the difficulty of follow-up due to disperse distribution of inhabitation and feasibility of management, some of the newborns were selected to be followed up. In rural Tibet, farmers lived very scattered so it was difficult to follow the newborns living in the more remote area with higher altitude and inconvenient transportation on time. According to the research plan, two townships with better ability of Maternal and child health management were selected from each county. In these townships, the infants born in the project were followed as far as possible. However, the infants selected to be followed presented similar background characteristics compared with the remaining infants including birth weight and length, parental educational level and family economic status, according to statistical analysis.

### Follow-up and anthropometry

Total 1418 pregnant women had normal delivery with live infants, of whom 253 were selected for following-up from their birth to 12^th^ month between August of 2010 and December of 2012. Five visits during the follow-up were conducted at their birth, 1^st^ month, 3^th^ month, 6^th^ month and 12^th^ month, respectively. Each visit occurred at the required visiting date (±10 days). Anthropometry of the subjects, as well as information on their feeding and immunization practice was collected at each visit. And the demographic and socioeconomic information of their families were ascertained at birth.

Anthropometry data on Tibetan infants and their mothers were collected by the trained maternal and child health care (MCH) staff. The infants’ birth weight and length were measured and recorded from the hospitals in which they were born. From 1^st^ month to 12^th^ month after birth, trained staff went to the infants’ home for measuring the weight and length. Infant recumbent length was measured in light clothes and to the nearest millimeter by using the infantometers (Xi’an Teaching Instrument Company, Model WB-II, precision 1 mm). Weight was collected by using an electronic infant scale (TANITA Corporation , BD-585, precision 100 g) when the infants were barefooted and bareheaded. Average weight and length from double measurements were used in analysis. The mother’s weight and height was measured only once at the baseline visit to the nearest millimeter by using adults’ boards (Tanita HD-305, Tanita Corporation, Shanghai, China). Ponderal Index (PI) was used here to measure the soft tissue growth of the infants and expressed as weight (kg)/length(m)^3^.

### Other variables

There were other variables used in the data analysis. The family demographic and socioeconomic information were ascertained at baseline including parental age and education, family size and economic status and so on. A face-to-face interview was conducted for the mothers and their children to collect related data on child health care, child morbidity, feeding information and mother’s prenatal care and so on at each visit. A designed questionnaire was used for data collection. When necessary, the fathers were asked to help the mothers provide family background information. All information was collected by a trained MCH staff. The mother was underweight as Body Mass Index (BMI) <18.5, normal as 18.5 ≤ BMI < 24 and overweight or obesity as BMI ≥ 24. Household wealth index for infants’ family was established from durable consumer goods of households including bicycle, motorcycle, washing machine, livestock and poultry, orchard and characters of house including types of house and toilet and floor materials using Principal component analysis. Four components were retained which cumulative contribution was 73.8%. Wealth index of each family obtained by contribution rate of each component multiplied variables above. Then the family’s living standard was categorized into rich, middle, poor according to their descending value of wealth index at three equal cutoffs.

### Quality control

The interviewers (the staff from local MCH stations) who can communicate freely in both Tibetan native language and Mandarin were trained and questionnaire administration and anthropometric measurements were standardized before the follow-up. During the follow-up, a checking system was applied including checking in the field by interviewer themselves, checking by officials in the counties and then by supervisor in the project office in Lhasa. Personnel from local hospitals and health offices assisted in organization of investigation and explanation of procedures to subjects. Subjects were re-interviewed when transcription or logical questions and missing values were found. Apparatuses for measurement were checked and/or adjusted for accuracy before daily work. All subjects were interviewed face to face to collect anthropometric data.

The study was conducted in accordance with the *Declaration of Helsinki* and was approved by Ethics Committee in Medical Research, Xi’an Jiaotong University (No. 20070712). A written informed consent to participate in the cohort study was obtained from each mother of the infant.

### Statistical analysis

Database was established using Epi data 3.1 software (The EpiData Association, Odense, Denmark) and duplication was adopted for data entry. Percentage was used to describe categorical variables. Mean and standard deviation (*s*) or median was used for description of continuous variables. The *t*-test and *χ*^*2*^ test were performed for comparative analysis. The longitude data on weight and length from the infants in Bolivia (3600 m) and Dutch were used as comparisons. Consideration of longitude data and its strong tolerance to missing values, mixed model was adopted to analyze the change of growth from the birth to 12^th^ month. Two mixed models were established. Firstly, a mixed model was used to explore the change of growth by gender among Tibetan children. In this model, the increment of weight/length between each visit and the birth was regarded as dependent variable and independent variables were gender, age, as well as the interaction of gender and age. Birth weight and length as adjustment was also in the model. Subjects were regarded as random variable. The model was indicated as following:


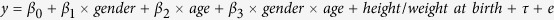


where *y* was the increment of weight/length, *β*_0_ was the intercept, *β*_1_ was coefficient of the gender, *β*_2_ was coefficient of the age, *β*_3_ was coefficient of the interaction of gender and *age*, *τ* was the random effects, and *e* was the standard errors. *Height/weight at birth* was controlled.

Secondly, in order to understand well the growth pattern of Tibetan infants, we further compared the difference in the growth pattern between Tibetan and Chinese Han infants by a mixed model with propensity scores (PS) technique. Data from 1388 Han infants used in this compared analysis was from a follow-up study conducted in two counties in western China, Bin and Changwu from 2003 to 2008^14^. The purpose of the study focused on maternal and child health care, and the same visiting methods was adopted as the follow-up study of Tibetan infants. PS technique along with the mixed model was employed when conducting the comparison in growth pattern between Tibetan children and Chinese Han children. Firstly we established the PS for each individual from two samples by logistic regression based on the socio-demographic factors, including parental age, education, occupation, as well as family size and household expenditure/year, which might affect the growth. When comparing the difference of growth pattern between the two groups, PS was used as a covariate in the mixed model as following:


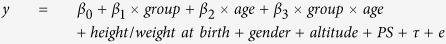


where *y* was the increment of both Tibetan and Han weight/length, *β*_0_ was the intercept, *β*_1_ was coefficient of the ethnic, *β*_2_ was coefficient of the age, *β*_3_ was coefficient of the interaction of ethnic and *age*, PS was the PS value of each infant, *τ* was the random effects, *e* was the standard errors. *Height/weight at birth, gender, altitude* and *PS* were controlled.

Moreover, PS was used as a matching factor, which meant that Tibetan and Han infants were matched by their PS and a matched subsample was used for the comparison in the growth pattern between them. As a sensitive analysis, a new mixed model was established, which was the same as the model above except no PS included. All the analysis was accomplished by Stata version 12.0 (StataCorp LP, College station 77845, USA). Significance of all hypothesis testing was determined at P < 0.05 with two-tailed.

## Results

### Characteristics of the sample of Tibetan children

There were 253 Tibetan newborns followed totally. Because of the geographical environment and traffic conditions in Tibet, some loss to follow-up happened at each visit. One hundred and eighty two infants were visited at 1^st^ month, 207 at 3^rd^ month, 240 at 6^th^ month, and 233 at 12^th^ month. The information of all the infants at birth was collected at the local hospitals. Of the sample, 129 were males and 124 females. The infants’ fathers were aged 27.4 years averagely (19.4~51.5 years old) and had 5.6 average schooling years. The mean age of their mothers was 26.5 years (17.5~43.5 years old) and the mean schooling years were 5.3 years. The mean height and weight of the mothers was 162.12 cm and 53.49 kg, respectively. About 15% of the mothers were underweight in early pregnancy. A Tibetan family had 6 members on average. Differences between both genders in the social-demographic characteristics were not statistically significant (*P* > 0.05) (Table 1).

### The change of weight and length with increasing age for Tibetan infants

The Table 2 showed the means in the weight and length of Tibetan infants from birth to 12^th^ month. The mean birth weight was 3.02 ± 0.51 kg, 3.07 ± 0.47 kg for male and 2.97 ± 0.55 kg for female. The weight were doubled at half a year after birth, and tripled at 12^th^ month. The average birth length of the infants was 48.33 cm, male 48.55 cm and female 48.12 cm, and the length increased by 15 cm after six months and by more than 23 cm after a year. The greatest increment for both weight and length occurred during the first month after birth and declined with increasing age. So did the PI increment. While not like weight and length, the highest value of PI was at 3^rd^ month then declined with month (Table 2).

Compared with Chinese Han infants and a group of European infants at low altitude, Tibetan infants and infants in Bolivia in high altitude had smaller measurements in both weight and length from birth to 12^th^ month, while they had a greater average increment in weight than Chinese Han and European infants at low altitude before 6^th^ month but their length increment did not show such trend (Table 2).

### Increment of weight and length of Tibetan infants from the birth

The Table 3 showed the increment of weight and height from the birth using the mixed model controlling for the weight and length at birth. In this model, socio-demographic characteristics were not adjusted because they were no significant difference between both genders, as showed in Table 1. The model indicated that the weight and length increment increased with age (*P* < 0.0001) regardless of male or female infants, but there was no gender difference found in the increment, and also there was no interaction between age and gender. The weight increment of male infants on average was 1.19 kg (95%CI: 0.95–1.43) at 1^st^ month and 6.28 kg (95%CI: 5.97–6.59) at 12^th^ month while it was 1.09 kg (95%CI: 0.88–1.31) and 6.28 kg (95%CI: 6.01–6.56) for female respectively. The length increment was 4.93 cm and 4.61 cm for male and female at first month and 23.61 cm and 24.23 cm at 12^th^month. Moreover, the birth weight/length had a negative impact on their increment after birth, which meant if an infant was longer or heavier at birth, it would have slower increment in length and weight (weight: *β* = −0.6819, *P* < 0.0001, length: *β* = −0.9571, *P* < 0.0001).

### Comparison of growth pattern between Tibetan and Chinese Han infants

**Figure 1:**
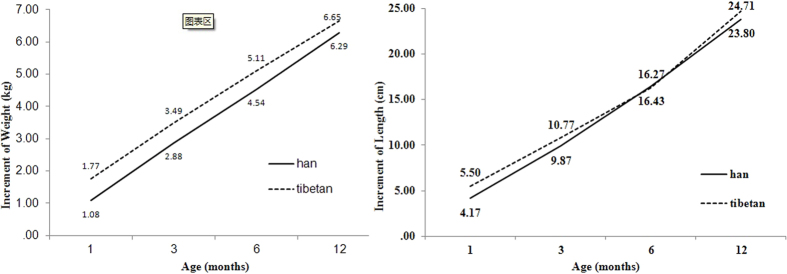
The estimated increments of weight/length from birth by age for Tibetan and Chinese Han infants from the mixed model adjusted for PS.

To compare the difference of growth pattern within 12^th^ month after birth between Tibetan and Chinese Han infants, the mixed model was established controlling for potential factors including gender, birth weight/length, altitude and PS. In the model, the interaction of ethnic and age was involved. The results of PS***-***adjusted model showed that the weight increment of Tibetan was greater than Han infant with age (*β*_age*ethnic_ = 0.0345, *P* < 0.001), while the length increment had no such trend (*β*_age*ethnic_ = −0.0212, *P* = 0.4385), after controlling covariant PS, gender, altitude and birth weight/length for both models (Table 4 and Fig. 1).

A sensitive analysis was conducted by changing analysis model. A subsample matched by PS was used for comparison in growth pattern of Tibetan infants with Chinese Han infants. After one-to-one match by PS, there were 244 children included in the new mixed model, half Tibetan and half Han. The results of PS-matched model indicated that the weight increment of Tibetan was greater than Han infant with age (*β*_age*ethnic_ = 0.0557, *P* = 0.0012), while the length increment had no such trend (*β*_age*ethnic_ = 0.0489, *P* = 0.3591), which were similar to the results from the PS***-***adjusted model (Table 4).

## Discussion

The smaller the age, the faster and easier the physical develops and pursues. The stage of infant lays the basis of the growth and development, further influences the production capacity of the adults^7^,^15^,^16^,^17^. There were few researches on evaluation of the nutritional status of Tibetan children living at high altitudes, especially based on a longitudinal study. Our study outlined the growth pattern of Tibetan infant by recording their weight and length at birth, 1^st^ month, 3^rd^ month, 6^th^ month and 12^th^ month, respectively. Characteristics of Tibetan infant’s growth within one year were obtained by using mixed model after controlling potential confounders. Differences of growth pattern between Tibetan and Chinese Han group were also analyzed to get a better understanding of ethnicity and altitude on infant growth. Our study showed that the fastest growth of Tibetan infants happened within the 1^st^ month after birth and there was no difference in genders in growth pattern within a year. The smaller the Tibetan infants were at birth, the faster they would grow up. Tibetan infants may have their own growth pattern, compared with Chinese Han infants or other ethnic infants.

### Basic feature of growth of Tibetan infants

The mean birth weight of Tibetan infants was 3.02 kg and grew by 0.54 kg per month on average. Meanwhile, their birth length was 48.33 cm and there were an increase of 1.93 cm per month averagely. The biggest increment of both weight and length occurred at first month after birth. The birth weight of the male was slightly higher than that of the female (3.07 kg v.s. 2.97 kg, *P* = 0.03), but there was no difference in birth height between male and female infants (48.55 cm v.s. 48.12 cm, *P* = 0.65). After controlling for the birth weight and length, the increment of weight and length of Tibetan infants increased rapidly with age. No interaction was found between age and gender, implying that the physical growth between male and female infants was similar in the first year of their life. This result was different from the Chinese research on Han children^8^,^18^. The birth weight and length had a significant influence on their later growth. This study also found that the increment of weight and length of Tibetan infants were negatively correlated with their birth weight and length. That means the smaller the measurements of weight and length at birth, the smaller their measurements of weight and length later, but their growth rate was greater than those with higher birth weight and length. This might imply that there was catch-up growth among the Tibetan infants with lower birth weight/height.

### Comparison in growth pattern of Tibetan infants with other ethnic infants

In this study, the growth pattern of Bolivia infants, Dutch infants and Chinese Han infants were compared with that of Tibetan infants. We found that Tibetan infants were smaller in both weight and length during their first year of life, compared with other ethnic infants at low altitude, like Chinese Han infants and Dutch infants^19^. However, their growth pattern was similar to their counterparts at high altitudes, such as Bolivia infants living in LaPaz with altitude of 3600 m^20^ and Tibetan infants living in Sherpas of Nepal^21^,^22^,^23^ because such infants living at high altitudes had greater increment of weight before 6^th^ month than their counterparts at low altitudes.

Further, in order to explore the features of growth pattern of Tibetan infants, we compared the physical growth between Tibetan and Chinese Han infants. These two follow-up studies were conducted in similar period and adopted the same visiting methods. The only difference was different subjects, i.e. Tibetan infants in rural Tibet and Chinese Han infants in rural Shaanxi. Therefore, we could observe ethnic difference in growth pattern between two groups of infants by controlling for potential confounding factors using mixed model with propensity scores technique. A significant finding was that Tibetan infants were smaller size at birth (lower weight and shorter length) compared with their Han counterparts living in plain of China and thereafter the weight and length of Tibetan infants at each visit was lower than that of Han infants. However, after controlling for potential confounders of socio-demographic factors by using PS as a co-variable in the mixed model, the weight increment of Tibetan infant was greater than Han infant with increasing age, but no such trend was found in length increment. We also adopted analysis of matched sample based on PS as a sensitive analysis. Results was similar to those from the mixed model adjusted for PS, which implied that although Tibetan infants had a lower birth weight, their increment was higher than Han infants within the first year after birth. This suggested that Tibetan infants were smaller in size at birth, but they had significant catch-up growth after birth, mainly weight. The increment of length of Tibetan infants was also increased with age, but showed no difference with Han infants. It might result from the shorter follow-up conducted in this study^24^,^25^. Moreover, our previous studies also found lower weight and length in the Tibetan children aged less than 3 years^7^,^26^. We suggested that Tibetan infants are born small size and might have a different growth pattern from other ethnic infants. Therefore, in the view of nutritional evaluation by anthropometry, WHO growth standards might not suitable to assess the nutritional status of Tibetan children because the high altitude might be an important and independent factor affecting the growth of Tibetan children^26^.

Ponderal index (PI), a measure of soft tissue growth, was involved as an indicator in our study. It reflected the mass of the body, both body fat and muscle mass, may have considerable prognostic value for subsequent growth and development of the newborn^27^,^28^. The lower value of PI might suggest a poor development of muscle and fat tissue in a negative uterus environment^29^. The PI value of Tibetan infants increased first and then decreased within their first year and reached the peak in 3^rd^ month, similar to the Han infants^18^. We also found the PI of Tibetan infants was higher than Han infants at each visit, including the birth PI, which implied that Tibetan infants were smaller but had a better body mass before and after birth. It might also be a sort of adaption to the highland environment. Study on maternal O_2_ transport suggested that the Tibetans may be better adapted as judged by less fetal growth retardation and may utilize maternal O_2_ transport mechanisms better than those living for a shorter time in highland^30^,^31^. A genome-wide allelic differentiation scan (GWADS) found eight single nucleotide polymorphisms (SNPs) located near EPAS1 in indigenous highlanders of the Tibetan Plateau (3,200–3,500 m) were observed at greatly elevated frequencies than closely related lowland Han, which encoded the transcription factor HIF2α and stimulated production of red blood cells and thus increased the concentration of hemoglobin in blood^32^,^33^. Pawson IG found that increased chest circumference, which seems to reflect a developmental acclimatization to hypoxia among Peruvian high-altitude natives, was not seen among the Tibetan in Sherpas^11^,^25^. Some researchers found that while hypoxia and other effects environmental agents did influence certain growth parameters, but genetic influences on growth may be more important among high altitude population^34^,^35^. Therefore, our study implied that Tibetan infants might have their own growth pattern, even within 1 year after birth, which might be outcome of adaption to higher altitude.

### Limitations

Although this follow-up study provided some significant information on the growth of Tibetan infants, there were some limitations considered carefully. Firstly, constrained by local traffics, language barrier and natural environment and funds, the sample size of the follow-up might be smaller to explore the general principle of the physical growth of Tibetan and further large-scale follow-up studies are required. Secondly, we just followed up the Tibetan children one year after birth and we did not know the growth pattern of these infants after 1 year. The effect of adaption to higher altitudes on the physical growth pattern of Tibetan children aged more than 1 year required further studies. Thirdly, in this study, we compared the growth pattern of Tibetan infants living at higher altitudes with that of Chinese Han infants living in the plain in order to explore the ethnic difference between Tibetan and Han infants. There were lots of factors affecting the growth of children, so we used mixed model to control for possible potential factors which were integrated into propensity scores, but there were still other unobserved confounding factors not included in this study. Moreover, this study did not provide gene study which was might be helpful to explain effect of the adaption to high altitudes on physical growth of Tibetan children.

## Conclusion

Although Tibetan infants had the lighter birth weight and shorter birth length, they had higher birth PI, which reflected a better body mass for Tibetan infants. Compared with Chinese Han infants, Tibetan infants were lower weight and shorter length within one year after birth but they had greater increment of weight which suggested that Tibetan infants had a significant catch-up growth with 1^st^ year of the life. The study implied that Tibetan infants had their own physical growth pattern different from Han infants and the adaption to highland environment could play an important role for the ethnic difference. Therefore, for comparison and assessment of physical growth status of Tibetan children, the effect of ethnicity on growth should be taken into account.

## Additional Information

**How to cite this article**: Wang, W. *et al.* The Growth Pattern of Tibetan Infants at High Altitudes: a Cohort Study in Rural Tibet region. *Sci. Rep.* **6**, 34506; doi: 10.1038/srep34506 (2016).

## Figures and Tables

**Table 1:** Socio-demographic characteristics of the Tibetan infants by gender.

Characteristics^1^	Male (*n* = 129)		Female (*n* = 124)		Total (*n* = 253)		***t/χ***^2^	***P***
	***n***	**%/** 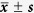	***n***	**%/** 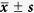	***n***	**%/** 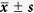		
Father’s age (year)								
<25	27	27.0	23	21.30	50	24.04	1.810	0.405
25~	41	41.0	54	50.00	95	45.67		
≥30	32	32.0	31	29.70	63	30.29		
Mother’s age (year)								
<25	55	44.35	50	42.74	105	43.57	0.081	0.960
25~	44	35.48	42	35.90	86	35.68		
≥30	25	20.16	25	21.37	50	20.75		
Mother’s height (cm)	129	162.36 ± 5.53	124	161.99 ± 5.84	253	162.12 ± 5.73	0.778	0.437
Mother’s weight (kg)	129	53.56 ± 5.59	124	53.43 ± 5.69	253	53.49 ± 5.64	0.453	0.651
Mother’s BMI (Kg/m^2^)^2^								
Underweight	17	14.91	21	18.75	38	16.81	2.959	0.226
Normal	95	83.33	85	75.89	180	79.65		
Overweight or obesity	2	1.75	6	5.36	8	3.54		
Mother’s education (year)								
0	27	21.09	34	27.42	61	24.21	6.589	0.086
1~6	41	32.03	50	40.32	91	36.11		
7~9	56	43.75	35	28.23	91	36.11		
≥10	4	3.13	5	4.03	9	3.57		
Father’s education(year)								
0	20	15.63	25	20.16	45	17.86	1.240	0.743
1~6	56	43.75	55	44.35	111	44.05		
7~9	49	38.28	42	33.87	91	36.11		
≥10	3	2.34	2	1.61	5	1.98		
Family size (persons)								
≤3	30	23.44	26	20.97	56	22.22	3.682	0.159
4~6	74	57.81	62	50.00	136	53.97		
≥7	24	18.75	36	29.03	60	23.81		
Living standard^3^								
poor	38	29.71	46	37.13	84	33.33	2.747	0.253
middle	42	32.84	43	34.74	85	33.73		
rich	48	37.45	35	28.23	83	32.94		

^1^For some variables, counts do not total 253 and percentages do not total 100% due to missing values.

^2^Mother’s BMI: BMI < 18.5 underweight,18.5 ≤ BMI < 24 normal, BMI ≥ 24 overweight or obesity.

^3^Living standards was measured by wealth index.

**Table 2:** The weight, length and Ponderal Index (PI) of Tibetan and other ethnic infants from birth to 12^th^ month ( 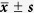).

	Ethnicity	Gender	Birth	1^st^ month	3^rd^ month	6^th^ month	12^th^ month	Relative increment per month (%)		
								0–3	3–6	6–12
Weight (kg)	Tibetan	Male	3.07 ± 0.47	4.26 ± 1.06	5.96 ± 1.22	7.68 ± 1.40	9.34 ± 1.36	31.38	9.62	3.60
		Female	2.97 ± 0.55	4.06 ± 0.98	5.88 ± 1.21	7.43 ± 1.39	9.25 ± 1.39	32.66	8.79	4.08
		Both	3.02 ± 0.51	4.16 ± 1.02	5.92 ± 1.21	7.55 ± 1.40	9.29 ± 1.37	32.01	9.18	3.84
	Chinese Han	Both	3.19 ± 0.41	4.96 ± 0.74	6.69 ± 0.88	8.30 ± 1.02	9.83 ± 1.23	36.57	8.02	3.07
	Bolivia (3600 m)^20^	Male	3.02 ± 0.37	3.86 ± 0.60	5.81 ± 0.58	7.59 ± 0.79	9.14 ± 1.11	30.79	10.21	3.40
		Female	3.01 ± 0.40	3.81 ± 0.51	5.61 ± 0.83	7.46 ± 1.13	9.04 ± 1.11	28.79	10.99	3.53
	Dutch^19^	Male	3.61 ± 0.50	4.57 ± 0.54	6.40 ± 0.68	8.09 ± 0.82	10.21 ± 1.01	25.76	8.80	4.37
		Female	3.47 ± 0.46	4.28 ± 0.49	5.83 ± 0.62	7.45 ± 0.76	9.53 ± 0.99	22.67	9.26	4.65
Length (cm)	Tibetan	Male	48.55 ± 3.89	53.48 ± 4.13	59.19 ± 4.29	65.28 ± 4.02	72.16 ± 4.51	7.31	3.43	1.76
		Female	48.12 ± 3.81	52.73 ± 3.33	58.28 ± 3.92	64.77 ± 4.51	72.35 ± 5.17	7.04	3.71	1.95
		Both	48.33 ± 3.85	53.12 ± 3.77	58.74 ± 4.13	65.02 ± 4.28	72.62 ± 4.88	7.18	3.56	1.95
	Chinese Han	Both	49.10 ± 2.48	54.59 ± 2.48	59.89 ± 2.41	65.32 ± 2.75	73.77 ± 2.76	7.33	3.02	2.16
	Bolivia (3600 m)^20^	Male	49.1 ± 1.60	52.2 ± 1.86	60.0 ± 2.22	64.8 ± 2.56	73.2 ± 2.08	7.40	2.67	2.16
		Female	48.2 ± 1.65	52.1 ± 1.61	58.9 ± 2.01	63.3 ± 2.30	72.3 ± 2.32	7.40	2.49	2.37
	Dutch^19^	Male	—	55.2 ± 2.0	62.0 ± 2.0	68.4 ± 2.1	76.5 ± 2.4	—	3.44	1.97
		Female	—	54.1 ± 2.0	60.4 ± 1.9	66.6 ± 2.0	74.9 ± 2.3	—	3.42	2.08
PI (kg/m^3^)	Tibetan	Male	27.68 ± 7.88	28.37 ± 10.09	28.86 ± 5.89	27.96 ± 5.43	25.35 ± 4.83	1.42	−1.04	−1.56
		Female	27.78 ± 8.70	27.91 ± 6.10	29.96 ± 6.55	27.68 ± 5.80	24.62 ± 5.45	2.62	−2.54	−1.84
		Both	27.73 ± 8.28	28.15 ± 8.37	29.40 ± 6.23	27.82 ± 5.62	24.95 ± 5.18	2.01	−1.79	−1.72
	Chinese Han	Both	27.10 ± 3.95	30.52 ± 3.82	31.14 ± 3.56	29.82 ± 3.53	24.51 ± 2.79	4.97	−1.41	−2.97

**Table 3:** The increment of weight (kg), length (cm) and Ponderal Index (PI) from birth by gender for Tibetan infant (95% Confidence Interval)^1^.

	Gender	*D*1	*D*2	*D*3	*D*4	Fixed effects (*β*)								Random effects (*β*)
						Gender	*P*	Month	*P*	Gender × Month	*P*	Birth value	*P*	Individuals
Weight	Male	1.19(0.95, 1.43)	2.89(3.62, 3.16)	4.61(4.32, 4.90)	6.28(5.97, 6.59)	0.1180	0.6040	1.6900	<0.0001	−0.0019	0.9816	−0.6819	<0.0001	0.8068
	Female	1.09(0.88, 1.31)	2.92(2.64, 3.19)	4.60(4.17, 4.75)	6.28(6.01, 6.56)									
Length	Male	4.93(3.79, 6.08)	10.64(9.50, 11.78)	16.73(15.66, 17.81)	23.61(22.39, 24.82)	1.4700	0.0790	6.5271	<0.0001	−0.4408	0.1479	−0.9571	<0.0001	11.2144
	Female	4.61(3.54, 5.67)	10.16(9.07, 11.25)	16.64(15.53, 17.76)	24.23(22.96, 25.49)									
PI^3^	Male	1.07(−0.33, 2.47)	2.50(0.47, 4.53)	1.37(−0.54, 3.29)	0.03(−2.00, 2.05)	0.9321	0.5373	−0.5503	0.4586	−0.4833	0.2988	−0.8710	<0.0001	10.5615
	Female	0.67(−1.76, 3.10)	3.31(0.995, 63)	0.94(−1.26, 3.15)	−2.50(−4.98, −0.01)									

^1^A mixed model was used here, in which dependent variables were the increments of weight, length or PI from birth and gender and month were fixed variables and interaction between them was included. Individuals were regarded as random variable and weight and length at birth were controlled.

^2^*D*1 to D4: the increment of measuring value at the 1^st^, 3^rd^, 6^th^ or 12^th^ month from birth, respectively.

^3^PI (Ponderal Index) = weight (kg)/length (m)^3^.

**Table 4:** Comparison of the growth pattern of Tibetan and Chinese Han infants by mixed models^1^

	*β*	*SE*^4^	*t*	*P*
**PS-adjusted model**^2^				
Weight increment				
Ethnicity	−0.1759	0.12092	−1.45	0.146
Month	0.4109	0.00319	128.45	<0.001
Month × Ethnicity	0.0345	0.00863	4.00	<0.001
Length increment				
Ethnicity	−1.7821	0.84410	−2.11	0.0349
Month	1.7338	0.05193	33.39	<0.001
Month × ethnicity	−0.02120	0.02736	−0.77	0.4385
**PS-matched model**^3^				
Weight increment				
Ethnicity	−0.3624	0.2532	−1.43	0.1538
Month	0.4078	0.0112	36.27	<0.0001
Month × Ethnicity	0.0557	0.0171	3.24	0.0012
Length increment				
Ethnicity	−0.4110	0.8307	0.49	0.6212
Month	1.6835	0.0343	49.04	<0.0001
Month × Ethnicity	0.0489	0.0532	0.92	0.3591

^1^The two mixed models were used here for comparison of weight or length increment between two ethnics (Tibetan and Chinese Han) and the reference for variable of ethnic was Han. PS referred to propensity scores.

^2^In the PS-adjusted model, the weight or length increment of Tibetan and Chinese Han children were compared controlling for gender, altitude, birth weight or length and PS.

^3^In the PS-matched model, the Tibetan sample and Chinese Han sample were matched with PS and then the weight or length increment of Tibetan and Chinese Han children were compared controlling for gender, altitude, birth weight or length.

^4^Standard error.
